# Immuno-Informatics Analysis of Pakistan-Based HCV Subtype-3a for Chimeric Polypeptide Vaccine Design

**DOI:** 10.3390/vaccines9030293

**Published:** 2021-03-21

**Authors:** Sajjad Ahmad, Farah Shahid, Muhammad Tahir ul Qamar, Habib ur Rehman, Sumra Wajid Abbasi, Wasim Sajjad, Saba Ismail, Faris Alrumaihi, Khaled S. Allemailem, Ahmad Almatroudi, Hafiz Fahad Ullah Saeed

**Affiliations:** 1 Department of Health and Biological Sciences, Abasyn University, Peshawar 25000, Pakistan; sajjad.ahmad@abasyn.edu.pk; 2 Department of Bioinformatics and Biotechnology, Government College University, Faisalabad 38000, Pakistan; farahshahid24@gcuf.edu.pk; 3 College of Life Science and Technology, Guangxi University, Nanning 530004, China; 4 Department of Medical, DHQ Hospital, Faisalabad Medical University, Faisalabad 38000, Pakistan; habiburrehman229@gmail.com; 5 NUMS Department of Biological Sciences, National University of Medical Sciences, Rawalpindi 46000, Pakistan; sumra.abbasi@numspak.edu.pk (S.W.A.); sajjadw@numspak.edu.pk (W.S.); sabaismail7@gmail.com (S.I.); 6 Department of Medical Laboratories, College of Applied Medical Sciences, Qassim University, Buraydah 51452, Saudi Arabia; f_alrumaihi@qu.edu.sa (F.A.); K.allemailem@qu.edu.sa (K.S.A.); aamtrody@qu.edu.sa (A.A.); 7 King Edward Medical University, Lahore 54000, Pakistan; fahadullah008@gmail.com

**Keywords:** hepatitis C virus, immuno-informatics, docking, molecular dynamics simulation

## Abstract

Hepatitis C virus (HCV) causes chronic and acute hepatitis infections. As there is extreme variability in the HCV genome, no approved HCV vaccine has been available so far. An effective polypeptide vaccine based on the functionally conserved epitopes will be greatly helpful in curing disease. For this purpose, an immuno-informatics study is performed based on the published HCV subtype-3a from Pakistan. First, the virus genome was translated to a polyprotein followed by a subsequent prediction of T-cell epitopes. Non-allergenic, IFN-γ producer, and antigenic epitopes were shortlisted, including 5 HTL epitopes and 4 CTL, which were linked to the final vaccine by GPGPG and AAY linkers, respectively. Beta defensin was included as an adjuvant through the EAAAK linker to improve the immunogenicity of the polypeptide. To ensure its safety and immunogenicity profile, antigenicity, allergenicity, and various physiochemical attributes of the polypeptide were evaluated. Molecular docking was conducted between TLR4 and vaccine to evaluate the binding affinity and molecular interactions. For stability assessment and binding of the vaccine-TLR4 docked complex, molecular dynamics (MD) simulation and MMGBSA binding free-energy analyses were conducted. Finally, the candidate vaccine was cloned in silico to ensure its effectiveness. The current vaccine requires future experimental confirmation to validate its effectiveness. The vaccine construct produced might be useful in providing immune protection against HCV-related infections.

## 1. Introduction

Hepatitis C virus (HCV) is a serious public health concern and a major cause of chronic liver disease [1]. HCV is responsible for hepatitis C, human lymphoma, and liver cancer (hepatocellular carcinoma) [2]. HCV is a member of the family Flaviviridae (genus *Hepacivirus*). It is a single-stranded, enveloped, small, and positive-sense RNA virus (55–65 nm in size) [3,4]. The virus causes both acute and chronic infections and affects more than 180 million people each year [5]. Asymptomatic acute HCV is often associated with life-threatening conditions and 15–45% of patients clear up the virus spontaneously within six months. Chronic HCV infections are associated with liver cirrhosis in 15–30% of the patients [6]. This disease is very common and found worldwide with a prevalence of 2.3% and 15% in WHO Eastern Mediterranean and European Regions, respectively. HCV prevalence in other WHO regions ranges between 0.5% to 1.0% (https://www.who.int/news-room/fact-sheets/detail/hepatitis-c; accessed on 10 March 2021). The infection rate is high (>3.5%) in Middle East, North Africa, and Central and East Asia. While in Adeana, Caribbean, Oceania, South and Southeast Asia, Australasia, sub-Saharan Africa, and Eastern and Western Europe, the rate is intermediate (1.5% to 3.5%). In regions, like North America, Asia-Pacifica and Tropical Latin America, the rate is <1.5% [7]. A high variation of HCV disease has been noticed and can be elevated in a certain population [8]. This variability is highly dependent on the usage of drugs, used syringes, other equipment, and surgical instruments. Additionally, there are multiple genotypes/subtypes of HCV with a variable distribution by regions that can also contribute to the disease burden [9].

Genome sequence analysis showed eight major genotypes of HCV (1–8) [10,11]. Genotype characterization is not only limited to the type of therapy needed for clearance of the virus but also suggests the level and risk of disease progression [12]. The prevalence of HCV genotype 1 is very high and contributes almost 49% of the disease followed by genotype 3, 4, and 2 which is 17.9%, 16.8%, and 11.0%, respectively while for the remaining 5% hepatitis, genotypes 5 and 6 are responsible [13]. Among all genotypes, HCV type 3 is poorly understood in the pegylated-interferon (PEGIFN) era with the lowest sustained virologic (SVR) response [14]. In the time period between 2001 and 2011, the standard therapy for chronic HCV infection was a blend of PEGIFN and ribavirin (RBV) and is refereed as PEGIFN era [15]. HCV type 3 genotype is considered to be one of the most complex types to treat in low and middle-income countries with a higher risk of disease progression and death. [16,17]. Infection caused by genotype 3 has also been linked to a severe and higher incidence of hepatocellular carcinoma (HCC) [18]. Accelerated liver fibrosis due to genotype 3 has been studied according to a meta-analysis and thus needs a more effective treatment to combat the genotype 3 virulence in developing countries [19]. Pakistan being an underdeveloped country, no good health surveillance system [20], low income and poverty has the second largest HCV burden. One out of every 20 Pakistanis is infected with HCV, and subtype-3a is the most common [21]. It is mostly transmitted by community (ear nose piercing, barbering), health workers (used syringes, blood transfusion) and high drug users through injection [22].

The current therapeutic approach for the treatment of HCV includes co-administration of antiviral drugs, more frequently PEGIFN, and simeprevir or sofosbuvir with ribavirin [23]. However, these treatments are linked to several drawbacks, such as high costs, drug resistance, or lack of protection against recurrence [24]. A variety of vaccine candidates are also in the development phase, with some having progressed to human clinical trials, including those based on synthetic peptides, recombinant proteins (core protein subunits and envelope proteins), viral vectors (virosome) and DNA (plasmid) [25,26,27,28,29,30]. Peptide and DNA-based vaccine candidates are currently being tested in murine models [31,32,33,34,35]. However, no HCV approved vaccine available to date. Hence, an effective and safe vaccine is, therefore, urgently needed to curb the global HCV disease burden [36].

Because of discrepancies in HCV genomic sequences and distribution by geographical region, a specific HCV vaccine produced for the Pakistani population would be ineffective. Since HCV subtype-3a infects a large proportion of the Pakistani population, the number of patients enrolled in public and private hospitals is steadily growing [37]. Hence, a vaccine against HCV, specifically HCV subtype-3a, is currently required to cover the majority of Pakistan’s population. Immunoinformatics approaches are a good alternative to predict and design highly immunogenic HCV multi-epitope vaccine candidates [38]. Immunoinformatics is an interface between computer science and experimental immunology that is used to examine essential immunological knowledge hidden in the immune system. A considerable number of studies have now been conducted to understand an immunoinformatics-based anti-HCV vaccine design [39]. Immunogenic epitopes that can generate successful responses from B and CD4+, CD8+ T cells should be included in an appropriate HCV polypeptide vaccine [40]. This study aims to investigate and design a polypeptide immunogenic vaccine candidate targeting Pakistani HCV subtype-3a using available computational immunology and immune-informatics tools and servers. The findings may be useful for experimentalists in designing either strain-specific or a broad-spectrum vaccine against HCV.

## 2. Materials and Methods

**Figure 1:**
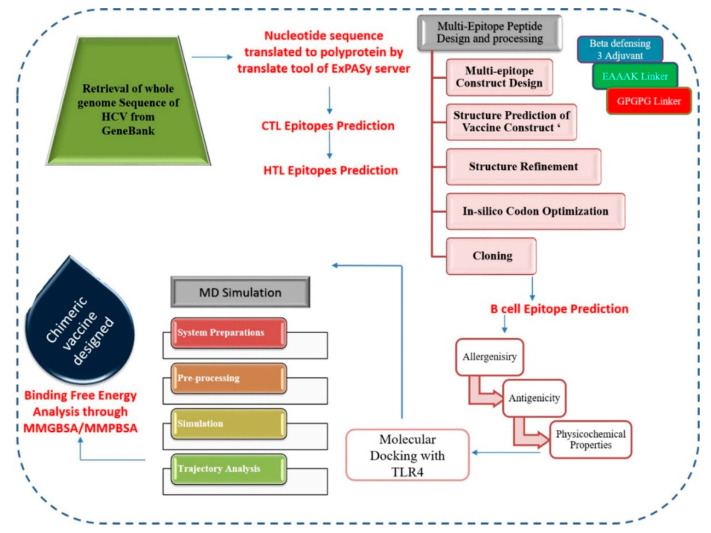
Graphical representation of the methodology employed for identification of potential antigenic epitopes in hepatitis C virus (HCV) subtype-3a.

All steps performed in this study for identification of antigenic peptides and formulation of chimeric polypeptide vaccine are presented in Figure 1.

### 2.1. Sequence Retrieval

The complete genome and polyprotein sequences of the Pakistani origin HCV subtype-3a were downloaded from the NCBI-GenBank database (Accession number: GU294484/PK) [41]. In-house Perl scripts were utilized for initial quality checking. The GU294484/PK genome sequence was chosen because of its validity, completeness, and quality. Next, the polyprotein sequence was subjected to helper T-lymphocyte (HTL) and cytotoxic T-lymphocyte (CTL) epitopes prediction.

### 2.2. CTL Epitopes Prediction

To obtain immunogenic CTL epitopes, HCV subtype-3a polyprotein was analyzed in NetCTL 1.2 server [42]. The viral protein was submitted in the FASTA format at the default threshold (0.75) for predicting CTL epitopes. From the predicted epitopes, all epitopes having a combined score of >0.75 were picked. The predicted epitopes were then submitted to the IEDB MHC-I binders predictor using default parameters [43]. Antigenicity, allergenicity, and IFN-γ checks were further performed on the predicted CTL epitopes to prioritize antigenic, non-allergenic, and IFN-gamma positive epitopes. For antigenicity, allergenicity, IFN-γ epitopes, VaxiJen [44], AllerTOP v. 2.0 [45] and IFNepitope servers [46] were used, respectively.

### 2.3. HTL Epitopes Prediction

The HTL epitopes for the HCV protein sequence were forecasted using the MHC-II epitope prediction module of the IEDB database with default parameters [43]. Generated epitopes were ranked on the basis of percentile rank. A lower percentile rank score signifies that HTL receptors have a high binding affinity. Like CTL epitopes, HTL epitopes were filtered based on antigenicity, allergenicity, and IFN-gamma inducing potential as described above.

### 2.4. Chimeric Polypeptide Vaccine Construction and Structure Prediction

To enhance the antigenicity of the individual epitope, a multi-epitope polypeptide was constructed. A linker, EAAAK, was used to link the Beta-defensin [47] (Q5U7J2_HUMAN) as an adjuvant to the end of the N-terminal of a construct, while AAY and GPGPG linkers were employed for joining epitopes. Polypeptide vaccine 3D structure was obtained by employing an online I-tasser server [48]. I-tasser is based on a pure ab-initio method to generate a 3D model without any homology template. The vaccine structure was refined through Galaxy Refine [49]. Ramachandran plot analysis was used to validate that refined structure.

### 2.5. Codon Adaptation and In-Silico Cloning

An increased rate of foreign gene expression within the host can be achieved through codon adaptation of the construct sequence, especially when the host’s codon usage is different from that of the source organism of the foreign gene. A codon that fails to adapt can result in lower rate of expression in the host. For this reason, the Java Codon Adaptation Tool (JCAT) [50] was employed to adapt the vaccine peptide construct’s codon usage to the *Escherichia coli* K12 strain. The adapted sequence was then cloned into vector pET28a (+) via the Snap Gene tool (https://www.snapgene.com/snapgene-viewer/; accessed on 15 February 2021).

### 2.6. B-Cell Epitopes Prediction

A complete sequence of the vaccine was subjected to B-cell epitopes prediction using Bepipred with default parameters [51].

### 2.7. Antigenicity and Allergenicity Prediction

To determine the ability of the vaccine to bind to T-cell and B-cell receptors resulting in stimulating immune responses and memory cell formation, ANTIGENpro was used [52]. The allergenicity of the vaccine was evaluated via AllerTOP v. 2.0 [45].

### 2.8. Physiochemical Properties Assessment

ProtParam web server [53] was further used to analyze physicochemical properties of the peptide including estimated half-life, theoretical pI, grand average of hydropathicity index (GRAVY), molecular weight (kDa), aliphatic index, and so on.

### 2.9. Molecular Docking and Dynamics Simulations

Molecular docking of the vaccine model was performed with TLR4 (PDB ID: 4G8A) using ClusPro 2.0 [54]. A simulation run of 100-ns was carried for the docked complex using AMBER18 [55]. System topologies were recorded using ff14SB [56]. The system was neutralized when 3 Na+ ions had been added and it was put in a TIP3P water box with a padding distance of 12 Å. Later, preprocessing was performed that can be divided into seven steps: first hydrogen atoms of the system were minimized for 500 cycles, then the water box was minimized for 1000 cycles. Non-heavy atoms were minimized for 300 cycles and alpha-carbon atoms were minimized for 1000 cycles with the restraint of 100 kcal/mol-Å2 and 5 kcal/mol-Å2, respectively. Moving ahead, the system was heated for 20 picoseconds (ps) at 300 K. To maintain temperature, Langevin dynamics [57] was used with a gamma value of 1. For constrain on hydrogen bonds, the SHAKE algorithm [58] was used, while for heating, a constant-temperature, constant volume (NVT) ensemble was employed. The system was then subjected to 100-ps with a time step of 2 femtoseconds (fs) in the pre-equilibration phase, while for pressure equilibration; the isothermal-isobaric (NPT) ensemble was used with 5 kcal/mol-Å2 restraint on alpha-carbon atoms. After this, with a restraint of 1 kcal/mol-Å2after every 10-ps, the pressure phase was extended for additional 50-ps. Lastly, the system was equilibrated for 1-ns. The total production run was 100-ns using the Berendsen algorithm with an NVT ensemble. For non-bonded interactions, an 8.0 cut-off was used, and the SHAKE algorithm was used for hydrogen atoms. Simulation trajectories were assessed using CCPTRAJ [59] of AMBER.

### 2.10. Estimation of MM/GB-PBSA Binding Energy

Binding free energy was computed for the simulated trajectories using MM/GB-PBSA methods [60,61]. From the trajectories, 100 snapshots were taken at regular intervals and the MM/GB-PBSA free energy difference was computed. The equation below was used for each snapshot to estimate the binding free energy, and the final energy was taken as the average score of all snapshots.

ΔGbinding = Gcomplex − (Gprotein + Gvaccine)

### 2.11. Immune Simulation

The C-IMMSIM server characterizes the immunogenicity and immune response of the designed vaccine [62]. This server is an agent-based model that predicts peptide interactions with the immune system using position-specific scoring matrices (PSSM). Two doses were given, with a four-week interval between them, and the simulation was run for 1000 steps.

## 3. Results

### 3.1. Sequence Retrieval

Genome sequence of Pakistani isolate of HCV subtype-3a (9474 bp) encodes a polyprotein (3027 amino acids) which was found to be non-allergenic and antigenic, with an antigenic score of 0.22 (ANTIGENPro) and 0.456 (VaxiJen). Further, CTL and HTL epitopes were predicted from this polyprotein.

### 3.2. CTL Epitopes Prediction

The CD8+ are the cytotoxic T-lymphocytes with a major role in T cell response against different pathogens like viral, bacterial, or protozoan infections [63]. In the case of any foreign particle, the antigen is presented by MHC-I with specific receptors on antigen-presenting cells (APCs). As a result of the immune response to the virus activation of different effector cells occur to kill or eliminate the antigen by different approaches. CTL epitopes were predicted through the NETCTL server. The immunogenicity of the polyprotein epitopes was analyzed according to the IEDB and NETCTL guidelines and only epitopes that showed a higher score and a much higher capacity to elicit the immune response were opted. Table 1 shows selected epitopes for the HCV polyprotein. In total, 54 epitopes were predicted; only 4 epitopes were filtered based on the good NETCTL and IEDB immunogenicity score. These epitopes are also antigenic, non-allergenic and IFN-γ inducing peptides.

### 3.3. HTL Epitopes Prediction

Besides cellular immune responses, a humoral immune response is also considered one of the key immune responses to different foreign particles or antigens [64]. Helper cells are considered to be one of the main contributors to the development of different prophylactics and immune-therapeutics [64]. Epitopes with a lower IC50 value will be ranked as highly immunogenic with more specificity and sensitivity to elicit a stronger immune response. In short, 5 different HTL epitopes with the lowest IC50 and a greater potential to initiate a good immune response were selected for vaccine design. Those epitopes which have intermediate or no affinity of binding to HTL alleles were not considered for downward analysis. Additionally, all these epitopes were found to have a greater potential for IFN-γ production which is a key contributor to viral immune response. The capacity for IFN-γ was obtained from its positive score using IFN epitope server output. The epitopes were also antigenic and non-allergenic. The HTL epitopes are presented in Table 2.

### 3.4. Chimeric Polypeptide Vaccine Construction

**Figure 2:**
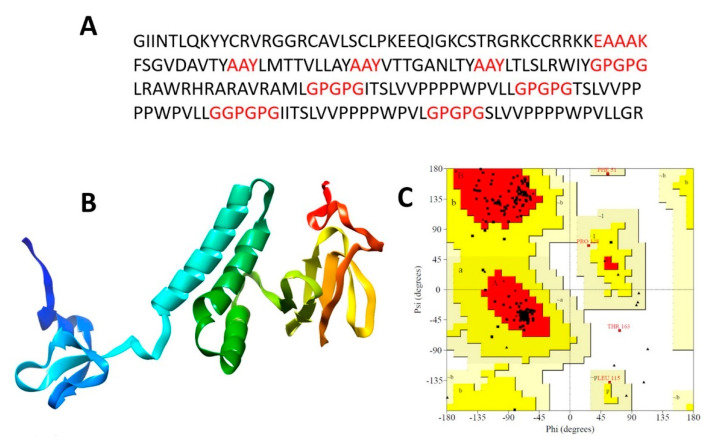
(**A**) Amino acid sequence of the designed polypeptide vaccine. The red sequence are linkers for fusing the epitopes. (**B**) Predicted 3D structure of the vaccine and (**C**) Ramachandran plot analysis of the vaccine.

A multi-epitope polypeptide vaccine was assembled by fusing all screened CTL and HTL epitopes (Figure 2A). Beta-defensin was used as an adjuvant molecule to improve the immunological response of the vaccine. The adjuvant was added to the N terminal site of the multi-epitope polypeptide using an EAAAK linker, allowing for the appropriate spacing of the functional domains as well as efficient expression and recognition by the host immune system. This was done for a reason as epitopes are weakly immunogenic therefore linking CTL and HTL epitopes via AAY and GPGPG linkers to attain maximum immunogenicity and expression of the epitopes results in the efficacy of the vaccine molecule. The final polypeptide consists of 195 amino acids.

### 3.5. Tertiary Structure Prediction, Refinement, and Validation

I-tasser server was employed to predict the vaccine candidate 3D model (Figure 2B). A total of 195 amino acid residues as a single domain with 1.5% disorder were modeled. From a secondary structure point of the view, the vaccine consists of 55% of helices, 39% coils, and only 4% of the beta-sheet. GalaxyRefine server for the protein refinement leads to enhance the number of residues in the favored regions. From an initial 87%, the number of residues in the Rama-favored region increased to 93% after refinement. The refined model was evaluated by Ramachandran plot and 2% residues in the outlier region, 5% residue in the allowed region, and 93% residue in the Rama-favored region were observed (Figure 2C).

### 3.6. In-Silico Cloning and Codon Adaptation

**Figure 3:**
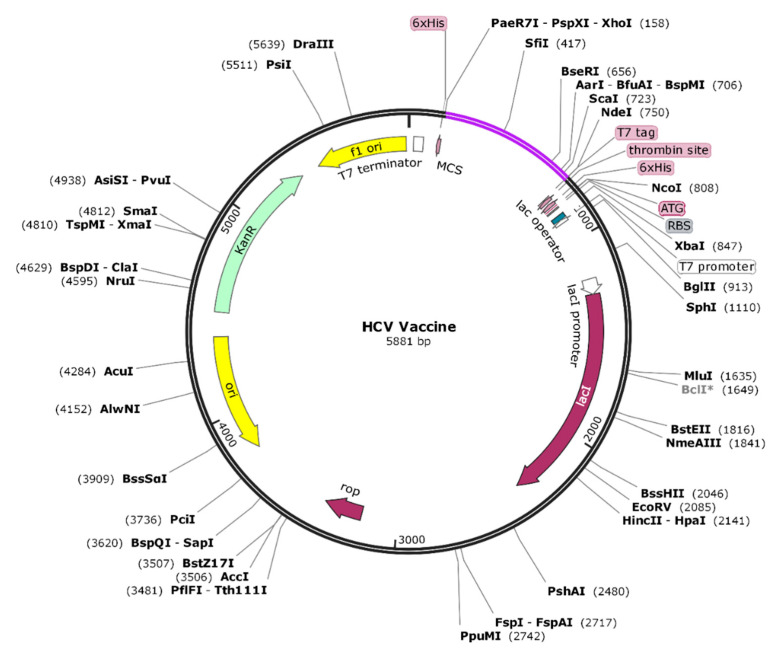
In-silico cloning of the vaccine (shown by indigo color) into pET28a (+) vector (shown by grey color).

The designed vaccine candidate was then cloned in-silico into a pET28a (+) vector for *E. coli* K-12 system expression. Prior to this, reverse translation of the vaccine amino acid sequence was done to optimize the sequence according to the usage of *E. coli*. This will ensure the good expression of the vaccine for experimental studies [65]. Codon optimization was done using the JCat server that gave a CAI (codon adaptive index) value of 0.9 and a GC content of 59.21% which are considered ideal in molecular biology for efficient protein expression. These values suggest vaccine proficient expression as the values are ideal as per the need in the *E. coli* system. The clone vaccine is presented via indigo color in the pET28a (+) vector given in Figure 3.

### 3.7. B-Cell Epitopes Mapping

B-cell epitopes play a significant role in stimulating humoral immune responses [66]. Epitopes responding to B-cell receptors (BCRs) are very crucial in vaccine design for antibody production. For accuracy and reliable predictability of B-cell epitopes, the IEDB server was used. The selection criteria were based on default cut-off score of 0.50. A total of 6 B-cell epitopes of variable length were predicted from vaccine candidate (Table 3). The presence of high score B-cell epitopes in the vaccine makes it a suitable candidate for development as it will enhance the humoral immune responses as well as cellular immunity.

### 3.8. Vaccine Antigenicity and Allergenicity

To evoke a significant humoral immune response, a successful vaccine candidate must be highly immunogenic. ANTIGENpro server was used to determine the antigenicity potential of the design vaccine construct, which was found to be 0.9, thereby representing the good potential of antigenicity. Moreover, allergenicity is one of the key factors in evaluating vaccine safety in humans, so it is important to predict its potential of causing allergic responses. AllerTOP, an online server, was used to analyze the vaccine allergenicity and predict the vaccine as non-allergic.

### 3.9. Vaccine Physicochemical Properties

ProtParam server was used to characterize different parameters including the molecular weight of the vaccine candidate. It was found that the vaccine had a 69.2 kDa molecular weight. The PI value of the vaccine molecule was 7.1 which confer that the vaccine will carry no charge at the mention pH value. The estimated half-life investigated in vitro of the vaccine construct in host cells was 31 h, while it is estimated to be >20 and >10 h in yeast and *E. coli*, in vivo. The extinction coefficient was calculated at 280 nm of 119,530 M^−1^cm^−1^ in water. The score for instability index was 39 that represents a stable and solid nature of vaccine construct. There is a direct relation between the higher value of the aliphatic index and thermal stability. The grand average and aliphatic index of hydrophobicity was 0.344 and thereby represent hydrophilic (because of negative value GRAVY) and thermostable nature.

### 3.10. Molecular Docking

**Figure 4:**
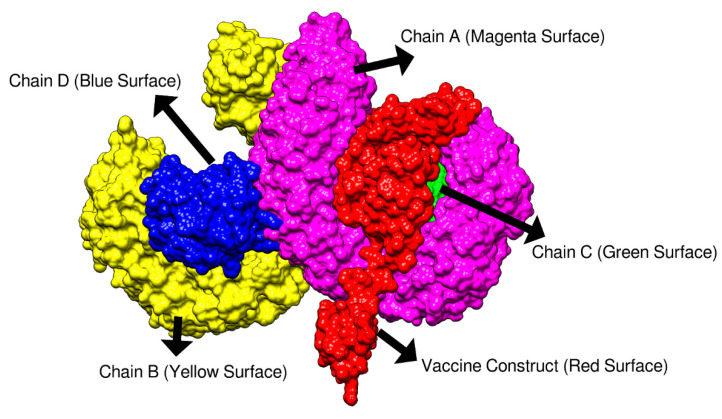
Docked conformation of the vaccine candidate to TLR4 molecule.

Molecular docking of TLR4 receptor with the vaccine candidate model was studied by ClusPro 2.0. Among the 30 models that were generated only the model with the lowest energy score and where the vaccine was properly engaged by the receptor was selected. Results revealed that the selected model number 1 accomplishes the desired criteria and considered the best-docked complex as shown in Figure 4. It was found that the dock complex (found to be −1187 kcal/mol) with the lowest energy score is in inverse relation with binding affinity. In our model we found a docked complex showing high binding affinity as the energy score was much lower. The vaccine molecule is engaged in the binding groove of chain A and chain C. From chain A, residues; Asn35, Glu286, Ser312, Arg355, Glu366, Asp379, Asp405, and Lys477 and from chain C, residues; Arg96, and Ser98 were determined to play a prime role in the binding of the vaccine within Å distance.

### 3.11. Molecular Dynamics Simulation

**Figure 5:**
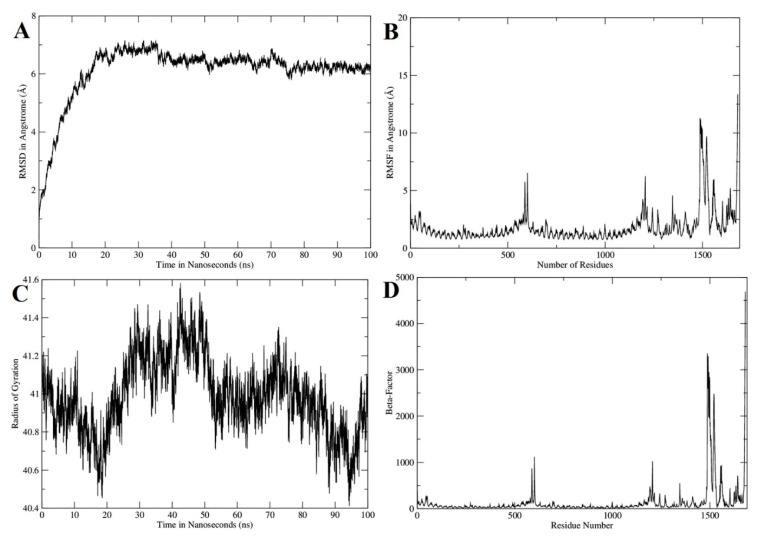
Different structural analysis based of molecular dynamics simulation trajectories. (**A**) RMSD, (**B**) RMSF, (**C**) Radius of gyration and (**D**) Beta factor.

The dynamics of the vaccine-TLR4 complex was explained through 100 ns of molecular dynamics simulation. As shown in Figure 5A, the initial 20 ns accounts for the adaption phase where the complex root-mean-square deviation (RMSD) was determined in a continuous surge. This high RMSD pattern may explain the sudden exposure of the complex to a dynamic environment allowing the complex to attain a proper conformation. Once a stable, conformation was achieved the system remained stable throughout the length of simulation time. To question the initial RMSD deviations, we further performed flexibility of residues of both TLR receptor and designed chimeric vaccine (Figure 5B). The majority of the residue level alterations can be noticed in the vaccine and upon trajectories, investigation loop regions were identified responsible for deviations which in turn cause higher RMSD of the system. Further, the system was subjected to the radius of gyration analysis and found that the system is not very compact therefore adding to higher gyration deviations (Figure 5C). This is in line with the first two analyses which depicted system deviation and in the same manner corresponding to the vaccine molecule loop regions flexibility. Anyhow, the intermolecular binding between the TRL4 and vaccine is strong and complex binding remained stable throughout the simulated time. The vaccine flexibility was unveiled by measuring the beta factor structural attribute (Figure 5D). This analysis also reported the same finding as demonstrated by the root mean square fluctuation (RMSF).

### 3.12. Estimation of MM/GB-PBSA Binding Energy

MM/GB-PBSA binding free energy estimation is an acceptable approach in determining the real efficacy of a ligand molecule to its receptor and highlighted key energies dominating complex formation. As can be pointed in Table 4, the vaccine binding to TLR4 is mainly dominated by van der Waals energy (ΔEvdW) as well equal significant contribution is reported from electrostatic forces (ΔEele). This signifies that both energies govern the stable binding of the vaccine to the receptor. On the other hand, polar solvation energy (Epolar.solv) is contributing less whereas non-polar solvation energy (Enon-polar.solv) has a positive impact during the binding process. Because of van der Waals and high electrostatic energy, the net gas phase energy is stable (−319.64 kcal/mol). The overall binding energy (ΔGbind(MM/GBSA)) of the complex is −282.2 kcal/mol.

### 3.13. Host Immune System Dynamics to Vaccine

**Figure 6:**
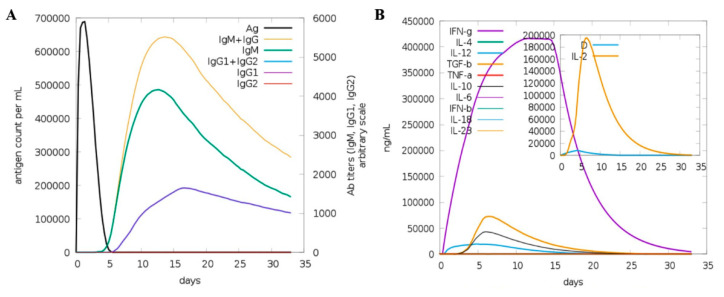
In silico immune simulation of the host in response to the vaccine antigen administration. (**A**) Antibodies titer to the vaccine, (**B**) interferon, cytokines and interleukins response to the vaccine.

The C-IMMSIM server was utilized to analyze the designed vaccine’s immunogenic profile. All the tertiary, secondary, and primary immune responses were significant contributors to the vaccine immunity. In particular, the combination of IgG + IgM antibodies were noticed in high titer, followed by IgM and IgG1 (Figure 6A). In addition to this, different B cell isotypes were formed in the response to vaccine administration resulting in memory cell formation. Additionally, the vaccine candidate induces high levels of IL-2 and IFN-γ (Figure 6B).

## 4. Discussion

HCV is a significant global public health issue as it can lead to hepatocellular carcinoma (HCC) and chronic liver disease [67]. Vaccination has proved to be the most efficient prophylactic method for maintaining public health and controlling infection spread. However, the manufacturing or efficient development of attenuated or live vaccines is costly and may take years to complete [68]. The inclusion of excessive antigenic load in the attenuated vaccine does, however, make the situation more difficult by causing allergic reactions and contribute little to the protective immune response. Multi-epitope vaccines remove adverse components that can lead to pathological immune response or adverse effects in comparison to the conventional vaccine [69]. Currently, several strategies for developing and designing competent and effective next-generation multi-epitope vaccines based on immunoinformatics approaches are readily available [70]. Previously, immunoinformatics-based approaches have been employed to propose efficient multi epitope vaccine candidates for Lassa virus [71], Zika Virus [46], Dengue virus [72], influenza virus [73], Middle East Respiratory Syndrome-Coronavirus (MERS-CoV) virus [74,75], Respiratory Syncytial Virus [76], Severe Acute Respiratory Syndrome-Coronavirus 2 (SARS-CoV-2) [77,78,79], chikungunya virus (CHIKV) [80] and many others. Because of the enormously reported merits and wide acceptability we adopted immunoinformatics-based approaches to design an effective vaccine candidate against Pakistani-based HCV subtype-3a that could efficiently enhance immune response after vaccination.

Genome and polyprotein sequence of Pakistani isolate of HCV subtype-3a was retrieved from GenBank. Antigenicity and allergenicity of polyprotein was evaluated and it was reported to be non-allergenic and antigenic. This provides an excellent start to map antigenic epitopes. Next, CTL and HTL epitopes were predicted from polyprotein sequence using various servers and databases. HTL activates both humoral and cellular immune responses, while CTL prevents the virus from spreading by secreting antiviral cytokines and killing virally infected cells. The identification of HTL and CTL epitopes is important for the designing of multi-epitope vaccines [81]. The vaccine candidate was designed by combining the CTL, and HTL epitopes with AAY, and GPGPG linkers respectively. Linkers are necessary elements of vaccines to improve folding, stabilization, and expression [82]. β-defensin was also attached as an adjuvant to the first CTL epitope via the EAAAK linker. Owing to the good antimicrobial and immunomodulatory properties, β-defensin acts as a very good adjuvant [83] and it has been used in many previous reported studies [84,85]. Multiepitope-based subunit vaccines need adjuvant coupling to enhance their immunogenicity [86]. In a vaccine formulation, adjuvants are additives that influence stability, some immune responses, durability, and growth of antigens and protect against infection [87]. Adjuvants are also advantageous to use because they allow the selective modulation of both humoral and cellular immune responses. The EAAAK linker is employed to join the adjuvant and epitope to enable efficient separation of the bifunctional fusion protein domains [88]. The designed vaccine with CTL and HTL epitopes along with adjuvant could have the potential to evoke neutralizing antibodies but also can inhibit the entry into the host cell, thus resulting in cell cycle arrest to inhibit the further spread.

The 3D structural modeling provides sufficient knowledge about the spatial arrangement of the main components of the protein. It also provides help in the study of protein functions and interactions with other proteins and ligands [89]. The desired properties of the vaccine construct were greatly improved after refining. The Ramachandran plot analysis represents 93% of residues in a favored region with 2% of residues in the disallowed region, showing the satisfactory quality of the model. Characterizing the physiochemical properties is vital while designing any potential vaccine. It was found that the vaccine had a 69.2 kDa weight which makes the vaccine easy to purify in the experimental evaluation. The PI value of the vaccine molecule is 7.1 which confer that the vaccine will carry no charge at the mention pH value. Instability index depicted that the protein has a stable nature upon expression, therefore augmenting the usage capacity further. The designed polypeptide is considered to be immunogenic, thermophilic, and highly hydrophobic in nature with reduced cysteine residues. All these physiochemical properties make this protein a suitable candidate as a HCV subtype-3 vaccine.

A stable connection is required between immune receptors (e.g., TLR-4) and candidate vaccine so that it can efficiently be transported within the host body [90]. HCV infection induces directly the expression of TLR4 and thus activates B cells, which can lead to innate immune responses of the host [91]. Molecular Docking analysis and MD Simulation not only confirmed the strong interactions between TLR4 and the vaccine construct but also demonstrated that a much lower amount of energy was needed for this stable binding in MMGBSA analysis. Minor fluctuations were observed during MD simulations. Hence, these results strongly suggest that the vaccine can proficiently bind to the immune receptors. Immunoreactivity testing through serological assessment is a fundamental step to authenticate a vaccine construct [92]. The translation of the foreign genes varies within the host system and the explanation behind this variation is the mRNA codon inconsistency; which requires codon optimization to ensure a higher degree of expression in the host cell [93]. GC content (59.21%) and CAI value (0.9) of optimized codons showed promising outcomes for higher protein expression in *E. coli* expression system, which are extensively used for the recombinant protein synthesis [94,95]. Next, it was analyzed that whether vaccine can stimulate the host immune system or not, after binding with the human immune receptor and transported into the body. The vaccine should hypothetically trigger cellular and humoral immune reactions. During immune simulation validation, our vaccine showed the highest production of IFN-γ, with significant IL-10 and IL-2 activities. Antibodies offer extracellular protection against HCV. There have also been noticed excess active immunoglobulins (i.e., IgG, IgM, and their isotypes which can be involved in the isotype switching). Besides, a variety of immune responses that can be thought of as a subunit vaccine contain multiple T-cell and B-cell epitopes are recommended by Simpson index.

In the current research, a vaccine candidate capable of producing immunological responses against HCV was designed. We assume that the designed vaccine will produce cell-mediated and humoral immune responses successfully. There were higher and stable binding patterns and interactions between vaccine and receptor. Additionally, successful immune reactions were noted in real life in immune simulation. Therefore, a vaccine carefully designed using such a technique may become an essential asset to combat viral infections. Immunoinformatics/Computing approaches were based on experimental methods to produce raw data for research purposes. The accuracy of immunoinformatics predictions will be limited by the consistency and efficiency of computer algorithms. Hence, in-vitro and in-vivo experiments are necessary to ensure the true potential of the designed vaccine to combat HCV.

## 5. Conclusions

HCV infection is a serious health issue concerning mortality and morbidity worldwide. No vaccine is available for the disease yet. In the current study, a chimeric polypeptide vaccine was designed based on the polyprotein sequence of a Pakistan-based HCV subtype-3a. Immunoinformatics and molecular docking approaches were used to design a latent and safe vaccine that can elicit cellular, humoral, and innate immune reactions. However, there are some limitations of the study that need to be investigated through experimental studies. For experimental studies, the real potency in protecting the host from infection is required to be performed in animal models. The reported vaccine candidate might be effective against HCV infection and therefore must be evaluated for prophylactic and therapeutic purposes.

## Figures and Tables

**Table 1:** Predicted cytotoxic T-lymphocyte (CTL) epitopes for HCV subtype 3.

Sr. No.	NETCTL	Score	IEDB Immunogenicity Score
1	FSGVDAVTY	2.044	0.1684
2	LMTTVLLAY	2.158	0.0949
3	VTTGANLTY	3.078	0.092
4	LTLSLRWIY	2.662	0.1325

**Table 2:** Predicted helper T-lymphocyte (HTL) epitopes for HCV subtype 3.

Sr. No.	IEBD (HTL Peptide)	Percentile Rank
1	LRAWRHRARAVRAML	0.17
2	ITSLVVPPPPWPVLL	0.29
3	TSLVVPPPPWPVLLG	0.29
4	IITSLVVPPPPWPVL	0.36
5	SLVVPPPPWPVLLGR	0.37

**Table 3:** Predicted B-cell epitopes in the vaccine construct.

Sr. No.	Start	End	Peptide	Length
1	24	50	LPKEEQIGKCSTRGRKCCRRKKFSGVD	27
3	102	105	VPPP	4
4	115	119	VVPPP	5
5	132	138	VPPPPWP	7
6	143	151	VVPPPPWPV	9

**Table 4:** Binding free energies of the vaccine candidate to TLR4.

Energy Component	Binding Free Energy (kcal/mol)
ΔEvdw	−234.48
ΔEele	−85.16
Epolar.solv	59.14
Enon-polar.solv	−21.7
ΔGgas (GBSA)	−319.64
ΔGsol (GBSA)	37.44
ΔGbind (GBSA)	−282.2

## Data Availability

The data presented in this study are available within the article.
